# Dominant changes in the breast muscle lipid profiles of broiler chickens with wooden breast syndrome revealed by lipidomics analyses

**DOI:** 10.1186/s40104-022-00743-x

**Published:** 2022-08-05

**Authors:** Ranran Liu, Fuli Kong, Siyuan Xing, Zhengxiao He, Lu Bai, Jiahong Sun, Xiaodong Tan, Di Zhao, Guiping Zhao, Jie Wen

**Affiliations:** Institute of Animal Sciences, Chinese Academy of Agricultural Sciences, State Key Laboratory of Animal Nutrition, Key Laboratory of Animal (Poultry), Genetics Breeding and Reproduction, Ministry of Agriculture, Beijing, China

**Keywords:** Broiler chicken, Lipid profile, Liver, Serum, Wooden breast

## Abstract

**Background:**

Chicken is the most consumed meat worldwide and the industry has been facing challenging myopathies. Wooden breast (WB), which is often accompanied by white striping (WS), is a serious myopathy adversely affecting meat quality of breast muscles. The underlying lipid metabolic mechanism of WB affected broilers is not fully understood.

**Results:**

A total of 150 chickens of a white-feathered, fast-growing pure line were raised and used for the selection of WB, WB + WS and control chickens. The lipids of the breast muscle, liver, and serum from different chickens were extracted and measured using ultra performance liquid chromatography (UPLC) plus Q-Exactive Orbitrap tandem mass spectrometry. In the breast, 560 lipid molecules were identified. Compared to controls, 225/225 of 560 lipid molecules (40.2%) were identified with differential abundance (DA), including 92/100 significantly increased neutral lipids and 107/98 decreased phospholipids in the WB/WB + WS groups, respectively. The content of monounsaturated fatty acids (MUFA) was significantly higher, and the polyunsaturated fatty acids (PUFA) and saturated fatty acids (SFA) were significantly lower in the affected breasts. In the liver, 434 lipid molecules were identified, and 39/61 DA lipid molecules (6.7%/14.1%) were detected in the WB and WB + WS groups, respectively. In the serum, a total of 529 lipid molecules were identified and 4/44 DA lipid molecules (0.8%/8.3%) were detected in WB and WB + WS group, respectively. Compared to controls, the content of MUFAs in the serum and breast of the WB + WS group were both significantly increased, and the content of SFAs in two tissues were both significantly decreased. Only five lipid molecules were consistently increased in both liver and serum in WB + WS group.

**Conclusions:**

We have found for the first time that the dominant lipid profile alterations occurred in the affected breast muscle. The relative abundance of 40.2% of lipid molecules were changed and is characteristic of increased neutral lipids and decreased phospholipids in the affected breasts. Minor changes of lipid profiles in the liver and serum of the affected groups were founded. Comprehensive analysis of body lipid metabolism indicated that the abnormal lipid profile of WB breast may be independent of the liver metabolism.

**Supplementary Information:**

The online version contains supplementary material available at 10.1186/s40104-022-00743-x.

## Background

In past decades, there has been a noticeable growth in the consumption of poultry meat as a result of health claims, the absence of cultural/religious prohibitions, and the efficiency of production [1]. However, the rapid increase in meat production has increased the occurrence of myopathies [2–4]. Wooden breast (WB) is one of the most serious myopathies adversely affecting meat quality [2]. Meat affected by WB is often marketed as a downgraded product, causing an estimated loss of $200 million per year in the United States [5]. Kuttappan et al. [6] demonstrated that around 85% of chicken breast meat showed WB at 9 weeks of age. Cruz et al. [7] estimated the occurrence of WB at up to 85.9% at 35 d and up to 89.2% at 42 d. The incidence of WB reached 60% in one big broiler processing plant in China [8].

WB myopathy is characterized by a significant increase in fat content and a decrease in protein and ash content [9, 10]. Lipids in skeletal muscle mainly include triacylglycerols (TGs) and phospholipids, which have significant effects on meat quality and processing characteristics [11]. The phospholipids are essential for the cell membrane and contain a high proportion (above 30%) of essential polyunsaturated fatty acids (PUFAs), The phospholipids with a higher polyunsaturated content than triacylglycerides are more susceptible to oxidation and therefore important for meat flavor development [12]. WB samples with higher fat have been reported in numerous studies [2, 9, 13]. However, despite their relevance, the lipid profiles of breast muscle exhibiting WB are quite limited.

WB begins progressively from the anterior end of the pectoralis major muscle and is often accompanied by white striping (WS) [14]. Phenotypically, WB can be detected by palpation [15] and a compression test [16]. Its histological changes mainly include moderate to severe polyphasic myodegeneration with regeneration and a variable accumulation of interstitial connective tissue or fibrosis [15]. It was reported that WB in broilers was related to metabolic perturbations [17]. Based on an examination of the results available in the literature, Soglia et al. [18] stated that the myopathic abnormalities in breast muscle have a complex etiology and several biological pathways and response mechanisms are involved in their progression. They summarized that the probable main involvement of sarcoplasmic reticulum stress and hypoxia in initiating the cascade of events resulted in the response mechanisms, including modifications in the energetic metabolism, inflammation, degeneration, and regeneration etc. In addition to breast muscle, liver metabolism might also be affected [19]. The level of serum creatine kinase (CK) has been found to have a significant correlation with the occurrence of WB [20]. These findings suggest that the progress of WB may be related to body metabolism.

With the development of analytical instruments and chemometrics, lipidomics have emerged as a subdiscipline of metabolomics [21]. Lipidomic analysis has been used in chicken meat to support food safety and authentication [22, 23]. Forty-seven typical lipid compounds were found in Taihe black-boned silky fowl by LC/MS-based lipidomics [24]. Lipid profiles in liver and serum were also studied [25, 26]. In the current study, the lipid profiles of breast muscle, liver and serum were studied using lipidomics approaches to clarify the lipid metabolic mechanisms of WB affected broilers.

## Methods

### Animals and sample preparation

A total of 150 chickens of a white-feathered, fast-growing pure line were raised by Xinguang Agricultural and Animal Industrials Co., Ltd. (Mile, China). All chickens were raised in three-story step cages with individual pens. The environmental and nutritional conditions were those recommended [27]. Diets and water were available ad libitum. The chickens were weighed individually at 42 days of age after fasting for 12 h. Blood samples were obtained from the wing vein and serum was separated by centrifugation (2500 r/min). All chickens were electrically stunned at 42 d. After slaughter, the chickens were immediately necropsied, and samples from the superficial layer of the cranial part of the right breast muscle were collected, snap-frozen in liquid nitrogen for downstream analysis. Breast muscle samples were also fixed in 4% paraformaldehyde for histological evaluation. The left breast muscle samples were deboned and stored in 4 °C for 3 h before scoring. Approximately 200 mg of liver sample was cut out and snap-frozen in liquid nitrogen and stored at − 80 °C for subsequent analysis.

### Muscle myopathy evaluation

All 150 breasts underwent WB scoring, and compression force measurements, respectively. WB was scored as described by Tijare et al. [28]. The subjective assessment included the absence of WB (normal breast score = 0), mild hardening in the upper part of the fillet (score = 1), moderate hardening in the upper and/or lower part of the fillet (score = 2), severe hardening (score = 3), and severe hardening plus hemorrhagic lesions, increased volume and/or presence of yellow fluid (score > 3). WB accompanied by WS was classified independently. The WS scores included no distinct white line (normal breast score = 0), moderate WS (small white lines, generally < 1 mm wide, but visible on the fillet surface, score = 1), severe WS (white lines 1–2 mm wide, very visible on the fillet surface, score = 2), and extremely severe WS (thick white bands > 2 mm wide, covering the entire surface of the fillet, score = 3) [29]. The compression force of fillets was measured as described by Sun et al. [16]. Fillets were measured in cranial regions and compressed to 20% with a 6-mm flat probe on a TA.XT Plus Texture Analyzer (Stable Micro Systems Ltd., Godalming, UK). The parameters were set as follows: trigger force was set at 5 g, probe height was 55 mm, pre- and post-probe speeds were 10 mm/s and the test probe speed was 5 mm/s. Each fillet was tested three times at different regions of the fillet. All chickens with moderate WB, which were top 10% of ranked scoring and the compression force, were selected as candidates for the next steps.

### Histopathological evaluation and serum creatine kinase determination

To confirm the affected groups, histological evaluations of breasts were conducted and the activity of serum creatine kinase (CK) was evaluated. The histological evaluations were mainly performed based on the method of Clark and Velleman [14]. Tissues were fixed in 4% paraformaldehyde for more than 24 h at 4 °C. Specimens were oriented transversally, dehydrated in a graded series of ethanol, and embedded in paraffin. Consecutive sections (6 μm thick) were prepared from each sample, and stained with hematoxylin and eosin. The myopathic lesions, such as myodegeneration and fibrosis, were assessed using a microscope (Olympus Corporation, Tokyo, Japan). The activity of serum CK was tested using a commercial kit (Nanjing Jiancheng Bioengineering Institute, Nanjing, China) following the kit protocol. Based on a previous report, serum CK activity was highly correlated with the occurrence of WB and the chickens with a CK value above 6.3 U/mL were selected for lipidomic analysis in the following lipid study [15, 20].

### Lipidomics analysis

A total of 42 breast, liver and serum samples from five controls, four chickens with moderate WB breast, and five with moderate WB + moderate WS were selected. The lipids were extracted and measured using ultra performance liquid chromatography (UPLC) plus Q-Exactive Orbitrap tandem mass spectrometry (Thermo Fisher Scientific, Waltham, MA, USA) according to the method reported by Tang et al. [30] with slight modifications. Tissue samples were mixed thoroughly. Approximately 65 mg tissue was added into 650 μL chloroform/methanol (2:1 v/v) and homogenized by a superfine homogenizer. Next 162 μL of water was added, vortexed for 2 min and incubated for 10 min at 4 °C, then centrifuged at 3000 r/min at 4 °C for 15 min. About 200 μL serum was added into 800 μL chloroform/methanol (2:1 v/v), vortexed for 2 min and incubated for 10 min at 4 °C, then centrifuged at 3000 r/min at 4 °C for 15 min. For the tissue and serum sample preparing, the isopycnic lower chloroform layer was transferred into a new tube (1.5 mL, Eppendorf, Wesseling-Berzdorf, Germany) and washed twice by aqueous liquid. The extracts were dried in a Thermo Scientific Savant Vac (Thermo Fisher, Waltham, MA, USA) for 1.5 h and the dry pellets were stored at − 80 °C. The pellets were dissolved and the protein concentration was measured and normalized to the protein level. Lipid extracts were obtained by a Cortecs C18 column (100 mm × 2.1 mm, Waters, Milford, MA, USA).

Untargeted lipidomics test was carried out with the Q-Exactive Orbitrap mass spectrometer coupled to a UPLC system Ultimate 3000 (Thermo Fisher Scientific, Waltham, MA, USA). The UPLC system was coupled to a Q-Exactive HFX Orbitrap mass spectrometer (Thermo Fisher Scientific, Waltham, MA, USA) which was furnished with a heated electrospray ionization (HESI) probe. A binary solvent system was utilized. The mobile phase A consisted of Acetonitrile (ACN): H_2_O (60:40), 10 mmol/L ammonium acetate, and the mobile phase B included iso-propyl alcohol (IPA): ACN (90:10), 10 mmol/L ammonium acetate. A 35-min gradient was applied with a flow rate of 220 μL/min. The sample tray and column chamber were preserved at 10 °C and 40 °C, respectively.

The data were obtained through data dependent acquisition by MS/MS. The mass spectrometer parameters were set as follows: a spray voltage of 3200 V (positive) and 2800 V (negative), an auxiliary gas flow rate of 10 Arb units, a capillary temperature of 320 °C, an NCE of 15/30/45, a mass range (*m/z*) of 240–2000 (positive) and 200–2000 (negative), and a topN of 10. A full scan and fragment spectra were acquired with a resolution of 70,000 and 17,500, respectively. Lipid identification was carried out by LipidSearch software (Version 4.1.16; Thermo Fisher, Scientific, Waltham, MA, USA). The individual lipid signal was obtained after the total signal was normalized. The relative quantification of the identified lipids was calculated from their relative peak areas.

### Statistical analysis

Statistical analyses were performed by SPSS 25.0 software (SPSS, Inc., Chicago, IL, USA). Statistical evaluation was carried out by the Student’s two-tailed *t*-test. Data are expressed as means ± standard error. Statistical significance was set at *P* < 0.05. Principal component analysis (PCA) and pathway analysis were performed using MetaboAnalyst 5.0. Graphs were generated with GraphPad Prism 8.0 software (GraphPad Software Inc., La Jolla, CA, USA).

## Results

### Different phenotypes in WB affected and normal chickens

Of the 150 chickens assessed, 37 with mild WB (24.7%), six with moderate WB (4%), 25 with moderate WB + moderate WS (16.7%), 62 with moderate WS (41.3%) and 39 normal (26.0%) were found by scoring. The WB affected group, including two sub-groups, moderate WB and moderate WB + moderate WS, were focused. As shown in Table 1, the WB scores in the WB and WB + WS group were 2.0. The WS scores in the WB + WS group were 1.0. The compression force and serum CK values in the affected groups were all significantly higher than that of controls.

**Table 1:** The breast muscle fillet indicators between the control and WB affected groups

Category	Control	WB	WB + WS
WB score	0	2.0	2.0
WS score	0	0	1.0
Compression force, N	2.57 ± 0.87	5.77 ± 1.00^***^	6.50 ± 0.45^***^
Serum CK, U/mL	5.39 ± 2.08	8.59 ± 2.01^**^	11.54 ± 1.83^***^

^**^P < 0.01; ^***^P < 0.001

**Fig. 1:**
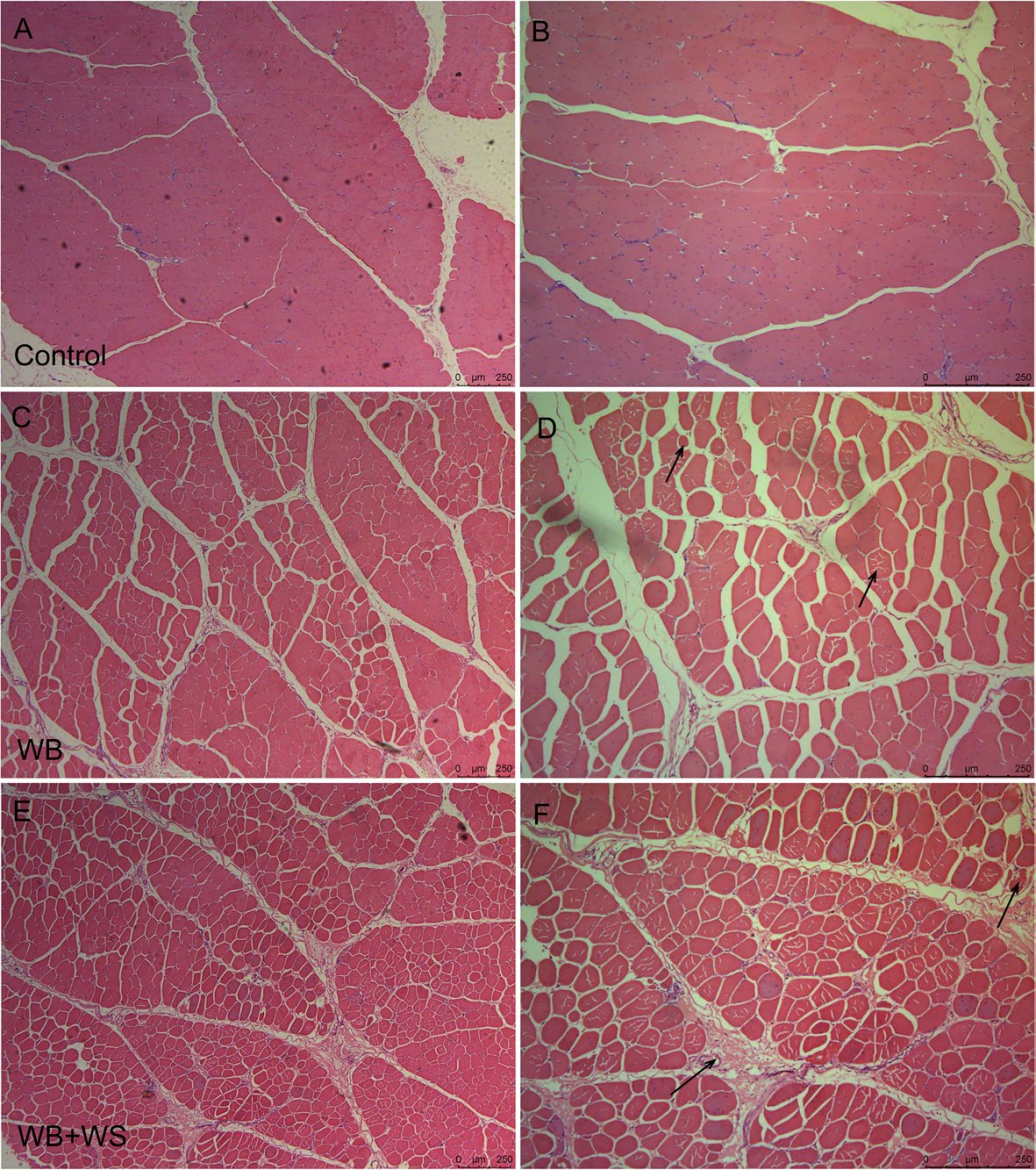
Histopathological observation of breast muscle in the control, WB and WB + WS groups. **A, B** The normal breast exhibited well-organized myofibers with a characteristic polygonal shape. **C, D** The WB group with irregular muscle cell morphology and myofiber degeneration and regeneration (arrows). **E, F** The WB + WS group with collagen deposition and dissolved myofibers (arrows)

As shown in Fig. 1, compared with the normal breast muscle (Fig. 1A, B), a wide variation in fiber size, multifocal myofiber degeneration, regeneration and dissolved myofibers were detected in the affected breasts (Fig. 1C, D, E, F). Degenerating fibers infiltrated by inflammatory cells and fibrosis characterized by diffuse collagen depositions were observed in the WB + WS group (Fig. 1E, F).

### The relative abundance of neutral lipids increased and phospholipids decreased in affected breasts

**Fig. 2:**
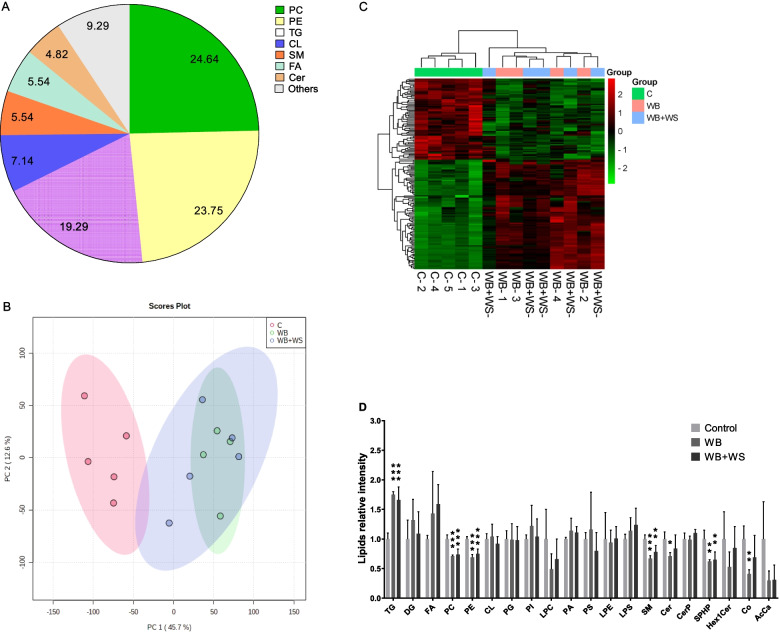
Lipid profiles in the control, WB and WB + WS chicken breast muscles. **A** Percentages of different lipid categories. PC, phosphatidylcholine; PE, phosphatidylethanolamine; TG, triglyceride; CL, cardiolipin; SM, sphingomyelin; FA, fatty acid; Cer, ceramides; PG, phosphatidylglycerol; PI, phosphatidylinositol; others included: CerP (1.25%), ceramide phosphate; LPC (1.07%), lysophosphatidylcholine; AcCa (0.71%), acyl carnitine; DG (0.54%), diglyceride; HexlCer (0.54%), simple Glc series; PA (0.54%), phosphatidic acid; PS (0.54%), phosphatidylserine; Co (0.36%), coenzyme; LPE (0.18%), lysophosphatidylethanolamine; LPS (0.18%), lysophosphatidylserine; SPHP (0.18%), Sphingosine phosphate. **B** Principal component analysis plot of lipidomics samples in the control, WB, and WB + WS groups. **C** Heatmap of significantly different lipid molecule categories affected by WB and WB + WS. Red cells and green cells in the heatmap represent increased and decreased lipids, respectively. **D** The relative differences in lipid content of different categories between the control and affected groups. LPE, LPS, PA, Cerp, PG, PI, CL, and FA were acquired in negative ion mode. Hex1Cer, AcCa, LPC, Cer, PS, SPHP, DG, Co, PE, SM, and TG were obtained in positive ion mode

The lipid profiles of control, WB and WB + WS breasts were determined. A total of 560 lipid molecules were identified and confirmed in positive and negative ion modes in the breast (Additional file 1). These lipids were further divided into 20 categories. The major lipid categories (Fig. 2A) were phosphatidylcholine (PC, 24.6%), phosphatidylethanolamine (PE, 23.8%), triglyceride (TG, 19.3%) and cardiolipin (CL, 7.1%). The control and affected groups could be distinguished in the PCA results (Fig. 2B). However, the WB and WB + WS groups could not be divided (Fig. 2B, C). The content of each lipid category was summed, respectively, and the differences between the control and affected groups were analyzed. Compared with the control group, the relative abundance of TGs significantly increased; however, the relative abundance of PCs, PEs, Sphingomyelins (SMs), Ceramides (Cers), Sphingosine Phosphates(SPHPs) and Coenzymes(Cos) significantly decreased in both the WB and WB + WS groups (Fig. 2D). Compared to the normal breasts, 225 differential abundance (DA) lipid molecules (40.2%) were detected in the WB group, including 97 increased and 128 decreased lipids. In the WB + WS breasts, 225 (40.2%) DA lipid molecules were also detected, including 116 increased and 109 decreased lipids (Table 2, Additional file 2).

**Table 2:** The numbers of differential abundance lipid molecules in different lipid categories of the WB affected breasts compared with the controls

Lipids/Groups	Neutral lipids		Phospholipids			Sphingolipid		Others	Total	Percentage, %
	TG	Others	PC	PE	Others	SM	Others			
WB									225	40.2
Up	92	–	–	–	–	1	4	1	97	17.3
Down	–	–	55	52	–	13	6	2	128	22.9
WB + WS									225	40.2
Up	95	5	1	2	1	5	7	–	116	20.7
Down	3	–	61	36	1	7	1	–	109	19.5

**Fig. 3:**
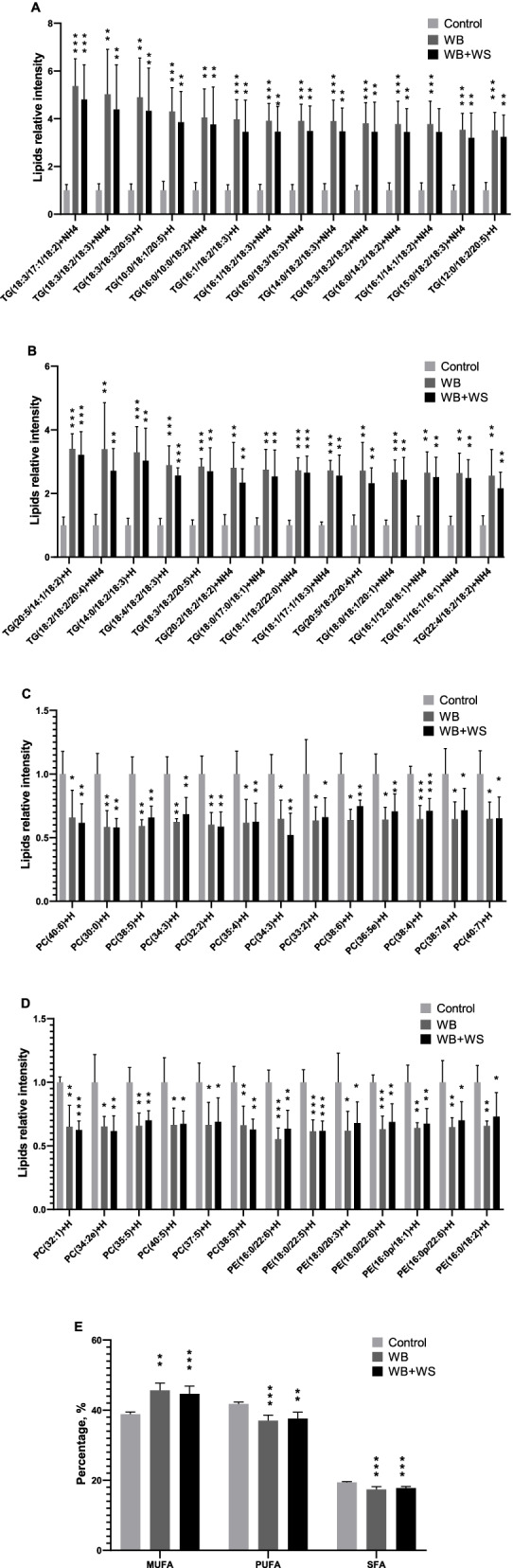
Shared differential abundance (DA) lipid molecules and the FA compositions in the control, WB and WB + WS chicken breast muscles. **A, B** The shared DA lipid molecules of TGs in affected groups with more than 2.5-fold change compared to the controls. **C, D** The shared DA lipid molecules of PCs and PEs in affected groups with less than 1.5-fold change compared to the controls. **E** Percentages of MUFAs, PUFAs, and SFAs in breast muscle FAs composition between the control and affected groups

In both the WB and WB + WS groups, the DA lipid molecules consisted of a dominant increased set of neutral lipids (92 in WB and 100 in WB + WS) and a decreased set of phospholipids (107 in WB and 98 in WB + WS) (Table 2, Additional file 2). When the results of DA lipid molecules in the WB and WB + WS groups were compared, there were 183 of 225 shared DA lipid molecules (*P* < 0.05, Additional file 2). The shared DA neutral lipids with abundance increased more than 2.5-fold compared to controls were shown in Fig. 3A-B. The shared DA phospholipids and sphingolipids with abundance decreased more than 1.5-fold were shown in Fig. 3C, D.

In the breast, the content of MUFAs in the controls was 38.9%, and the levels were significantly increased in the WB (45.6%) and WB + WS group (44.7%). For PUFAs, the levels were significantly decreased in the WB group (37.0%) and WB + WS group (37.6%) compared to controls (41.8%). For SFAs, the levels were significantly decreased in the WB group (17.4%) and WB + WS group (17.8%) compared to controls (19.4%). The varied trend in the fatty acid composition of glycerides was consistent with that of breast muscles between affected groups and controls. There was, however, no corresponding difference found in the fatty acid composition of phospholipids (Additional file 3) .

### Minor changes in lipid profiles in the liver and serum of the affected groups

In the liver, a total of 434 lipid molecules were identified (Additional file 4). Only 29 DA lipid molecules (6.7%) were detected in the livers from the affected groups compared with controls (Table 3). For the WB + WS group, 61 DA lipid molecules (14.1%) were detected (Table 3, Additional file 5). In the serum, a total of 529 lipid molecules were identified (Additional file 6). Compared with controls, four (0.8%) and 44 (8.3%) DA lipid molecules were detected in the WB and WB + WS group, respectively (Table 3, Additional file 7).

**Table 3:** The numbers of increased and decreased lipid molecules in different lipid categories of the WB affected group compared with the control group

Tissues	Lipids/Groups	Total	Percentage, %	Differential lipid molecules
Liver	WB	29	6.7	17 lipid molecules increased (Neutral lipids: 10, Phospholipids: 7) 12 lipid molecules decreased (Phospholipids: 6, Sphingolipid: 4;Others: 2)
	WB + WS	61	14.1	39 lipid molecules increased (Neutral lipids: 2, Phospholipids: 29; Sphingolipid: 8) 22 lipid molecules decreased (Phospholipids: 19, Sphingolipid: 3)
Serum	WB	4	0.8	3 lipid molecules increased (Sphingolipid: 3) 1 lipid molecules decreased (Sphingolipid: 1)
	WB + WS	44	8.3	31 lipid molecules increased (Neutral lipids: 6, Phospholipids: 3; Sphingolipid: 22) 13 lipid molecules decreased (Neutral lipids: 4;Phospholipids: 7; Sphingolipid: 2)

### Comparative analysis of the DA lipid molecules between the breast muscle, liver, and serum

**Fig. 4:**
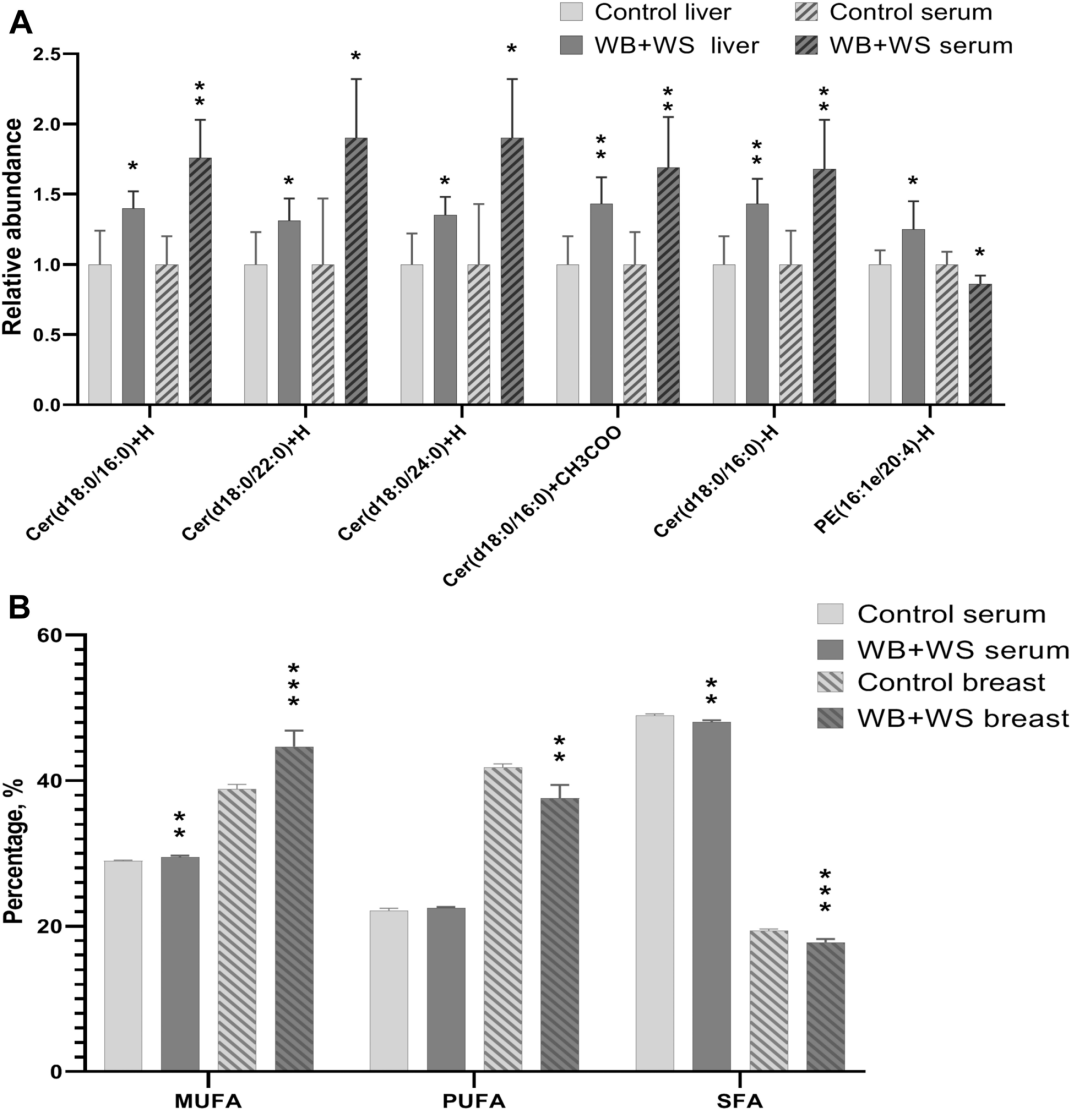
The shared differential abundance (DA) of lipid molecules between different tissues. **A** The shared DA lipid molecules of WB + WS groups between liver and serum. **B** The changing of FAs composition in breast muscle and serum when compared WB + WS groups to controls. MUFAs, monounsaturated fatty acids; PUFAs, polyunsaturated fatty acids; SFAs, saturated fatty acids (*P < 0.05, ** *P* < 0.01, *** *P* < 0.001)

For the comparative analysis between breast and serum, the changing pattern of FAs composition was detected. Consistently, the content of MUFAs in the serum and breast of the WB + WS group were significantly higher than the controls, respectively. The content of SFAs in the serum and breast of the WB + WS group were significantly lower than those in the controls (Fig. 4B). There was no consistent pattern change detected between those in the serum and breast when the WB group was compared with the controls.

For the comparative analysis between liver and serum, shared DA lipid molecules from those found between the affected groups and controls were screened. Six shared DA lipid molecules were detected between the WB + WS group and controls, of which five lipid molecules were consistently increased in both liver and serum, including Cer (Cer (d18:0/16:0) + H, Cer (d18:0/22:0) + H, Cer (d18:0/24:0) + H, Cer (d18:0/16:0)-H, and Cer (d18:0/16:0) + CH_3_COO). However, the relative abundance of PE (16:1e/ 20:4)-H, changed inversely between the liver and serum (Fig. 4A). There were no shared DA lipid molecules found in liver and serum when the WB group was compared to the controls.

## Discussion

WB myopathy is characterized by a significant increase in fat content [2, 9, 13]. In this study, chickens were selected strictly by breast palpation, WB and WS scores and compression force, and the serum CK level and tissue histological examination were performed for confirmation [15, 16, 20]. Lipidomics analysis showed that the abundant of 40.2% of lipid molecules changed in the affected groups, including a dominant increase in the abundance of neutral lipids (mainly TG) and a decrease in the abundance of phospholipids (mainly PE and PC). To our knowledge, this is the first report on the comprehensive lipid characteristics of broiler chickens with WB syndrome. Our finding of a dominant increase in the abundance of neutral lipids is consistent with previous reports on the fat increase in WB meat [2, 9, 13]. Chicken breasts with severe WS showed muscular cells with smaller areas and diameters (*P* < 0.05) [31]. Phospholipids are the main components of cell membranes [32]. The degeneration of muscular cells may be the result of the abundant decrease in phospholipids in affected breasts.

In this study, a higher level of MUFAs, and a lower level of PUFAs and SFAs were found in affected breasts. In a previous study, the SFA content was consistently found to be lower in WS breast compared to normal breast. However, few consistent results were found for MUFA and PUFA content changes in affected breasts [33, 34]. The development of WB or WS is closely related to oxidative stress of breast muscle [35]. At the molecular level, evidence for the exposure to free radical compounds, as depicted by the presence of lipid peroxidation products, has been observed in WS and WB [17, 31]. The decrease of PUFAs in affected breast found in the current study supports the occurrence of lipid peroxidation in WB fillets.

The progression of WB is complex, and its etiology may not be limited to the muscle alone, but related to altered systemic pathology [36]. Previous reports have shown that upregulation of the genes involved in lipid uptake and transport has been observed in 3-week-old breasts and marketable age broilers [37–40]. In the current study, the content of MUFAs in the serum and breast of the WB + WS group were significantly higher than those in the controls. The content of SFAs in the serum and breast of the WB + WS group were consistently lower than those in the controls. There was no consistent pattern detected between different tissues when the WB group was compared with the controls. It indicated that the changing of breast FA composition might be related to those of serum in WB + WS group. However, with regard to the lipids in liver, only 61 DA lipid molecules were detected in the WB + WS group compared with the controls, and only five DA lipid molecules were consistently changed between liver and serum. In the WB group, there was no consistent change detected between the different types of FAs in the serum and breast, and no shared DA lipid molecules were found in the liver and serum. The results indicated that the 40.2% of DA molecules found both in moderate WB and WB + WS breasts are unrelated to the minor changes in lipids in the liver [41–43]. Xing et al. [44] demonstrated that the liver of moderate to severe WB chickens showed oxidative stress, apoptosis and inflammation. In the future, body lipid metabolism of broilers with severe WB should be studied to clarify its relationship with the development of myopathy.

Previous research demonstrated that different types of lipids have different effects on volatile products [11, 45]. Intramuscular lipids, especially phospholipids, contribute significantly to pork flavor [46]. The lipolysis of phosphatidylethanolamine (PE) is the major contributor to the increase in free fatty acids, which are important for meat flavor in the processing of Nanjing dry-cured duck [47]. In this study, decreased phospholipids were found in affected breasts which might affect chicken flavor. The higher content of PUFA chains, the more likely they are to cause discoloration, off-odor formation and lipid oxidation products during processing and storage, which will reduce both meat shelf-life [48, 49]. It was demonstrated that the microbial shelf-life of WB and WS meat is longer than normal meat [34]. In the current study, higher levels of MUFAs, and lower levels of PUFAs and SFAs were detected in WB affected meat. The specific influence of changes in FAs composition upon meat processing of affected meat requires further investigation. Normally, degraded WB meat is made into processed products and it was reported that marination and cooking methods could not improve the quality and texture of WB and new methods need to be explored [50]. As the current demand for updated processing methods for WB meat is increasing, the alterations in lipid profile should receive attention to benefit WB meat processing.

## Conclusion

Our study found that the relative abundance of 40.2% of lipid molecules were changed in affected breasts compared to controls, including 92/100 increased neutral lipids and 107/98 decreased phospholipids in the WB and WB + WS groups. In the liver, the 6.7–14.1% of DA lipid molecules were detected in the affected groups. In the serum, the 0.8–8.3% of DA lipid molecules were detected in the affected groups. The content of MUFAs was significantly higher and SFAs and PUFAs were significantly lower in affected breasts. Compared to controls, the content of MUFAs in the serum and breast of the WB + WS group were both significantly increased, and the content of PUFAs and SFAs in two tissues were both significantly decreased. Only five lipid molecules were consistently increased in both liver and serum in WB + WS group. Taken together, these results revealed the dominant alterations in lipid profiles in moderate WB and suggested that these abnormal changes might be independent of liver metabolism. The findings in this study provide new knowledge on the lipid metabolic mechanism underlying WB myopathy.

## Supplementary Information

**Additional file 1.** The peak areas of all the lipid molecules identified in the breast of control and affected groups.

**Additional file 2.** Lipid molecules with significant different relative abundance in the WB and WB + WS breasts compared to the controls

**Additional file 3.** Alterations in fatty acid compositions of phospholipids and glycerides in WB affected breasts compared to the controls

**Additional file 4.** The peak areas of all the lipid molecules identified in the liver of control and affected groups.

**Additional file 5.** Lipid molecules with significant different relative abundance in the liver of WB and WB + WS groups compare to the controls.

**Additional file 6.** The peak areas of all the lipid molecules identified in the serum of control and affected groups.

**Additional file 7.** Lipid molecules with significant different relative abundance in the serum of WB and WB + WS groups compared to the controls.

## Data Availability

All data generated or analyzed during this study are included in this published article.

## Abbreviations

AcCa: Acyl carnitine
Cer: Ceramides
CerP: Ceramides phosphate
CF: Compression force
CK: Creatine kinase
CL: Cardiolipin
Co: Coenzyme
DA: Differential abundance
DG: Diglyceride
FA: Fatty acid
Hex1Cer: Simple Glc series
LPC: Lysophosphatidylcholine
LPE: Lysophosphatidylethanolamine
LPI: Lysophosphatidylinositol
LPS: Lysophosphatidylserine
MUFA: Monounsaturated fatty acid
PA: Phosphatidic acid
PC: Phosphatidylcholine
PCA: Principal component analysis
PE: Phosphatidylethanolamine
PG: Phosphatidylglycerol
PI: Phosphatidylinositol
PUFA: Polyunsaturated fatty acid
PS: Phosphatidylserine
SFA: Saturated fatty acid
SM: Spaghetti meat
SPHP: Sphingosine phosphate
TG: Triglyceride
UPLC-MS: Ultra performance liquid chromatography
WB: Wooden breast
WS: White striping
